# Managerial short-termism, firm-level financial instability, and socioeconomic determinants of health: evidence from Chinese listed firms

**DOI:** 10.3389/fpubh.2026.1826827

**Published:** 2026-07-23

**Authors:** Yun-Chung Li, Chia-Hsien Tang

**Affiliations:** 1 School of Oversea Education, Sanming University, Sanming, Fujian, China; 2 School of Economics and Management, Guangzhou Vocational and Technical University of Science and Technology, Guangzhou, China

**Keywords:** corporate governance, financial instability, managerial short-termism, real earnings management, socioeconomic determinants of health, stock price crash risk

## Abstract

**Introduction:**

Firm-level financial instability generates economic insecurity relevant to the socioeconomic determinants of health. Stock price crashes are an extreme form of such instability, yet prior work emphasizes information opacity rather than the managerial behavior that lets adverse information accumulate. We ask whether managerial short-termism raises firm-level crash risk.

**Methods:**

Using 27,856 firm-year observations from non-financial Chinese A-share firms (2009–2024, CSMAR), we treat real earnings management (REM) as an observable manifestation of managerial short-termism. Crash risk in year t+1 is measured by negative conditional skewness (NCSKEW) and down-to-up volatility (DUVOL) in fixed-effects models.

**Results:**

REM is positively associated with subsequent crash risk, consistently across alternative measures and robustness checks. Longer CEO equity incentive horizons and stronger analyst monitoring attenuate this association, which is stronger in essential-service industries.

**Discussion:**

Managerial short-termism is a behavioral source of firm-level financial instability that incentive design and information environments can mitigate. Because endogeneity cannot be fully excluded, the findings are robust associations rather than causal evidence.

## 1 Introduction

Large stock price crashes represent a severe form of firm-level financial instability. Unlike ordinary price fluctuations, crash events involve abrupt and substantial declines in stock prices that can erase a large portion of firm value within a short period. Such extreme downside events often emerge after prolonged periods of apparently stable performance and low volatility, during which firm-specific risks accumulate gradually. In many cases, the buildup of negative information becomes visible only after the crash occurs, suggesting that unfavorable news was not incorporated into prices in a timely manner.

Beyond their financial implications, episodes of firm-level financial instability may also generate broader socioeconomic consequences that are relevant for public health. Sudden declines in firm value and financial distress can affect employment stability, wage growth, and household economic security. From a health economics perspective, these economic disruptions may be related to socioeconomic conditions associated with health outcomes by increasing psychological stress, reducing access to healthcare services, and weakening household financial resilience.

A growing body of health economics research documents that economic instability and financial crises are associated with adverse health outcomes. Prior studies show that economic downturns can increase psychological distress, mental health disorders, and mortality risks (1, 2, 35). Financial shocks may also affect healthcare utilization and long-term wellbeing through income instability and reduced social protection (3). These findings suggest that financial instability may represent an important pathway through which economic conditions influence public health outcomes.

At the firm level, a large body of research explains stock price crashes through delayed information disclosure. When managers have discretion over reporting and disclosure decisions, they may postpone the release of unfavorable information. As a result, stock prices remain elevated until accumulated bad news is disclosed over a short period (4, 5). Empirical evidence shows that firms with opaque reporting practices or weak monitoring mechanisms tend to face higher crash risk. By contrast, stronger disclosure practices, improved governance structures, and greater external oversight are associated with lower crash risk (6–10).

Despite this evidence, an important question remains unresolved: why do managers continue to delay unfavorable information even when such behavior increases the likelihood of severe price declines? Most existing studies treat information opacity as an outcome of institutional settings, reporting standards, or governance structures. Less attention has been given to managerial behavior as a determinant of disclosure timing. This creates an important gap between the crash-risk literature and the managerial short-termism literature. The crash-risk literature explains how hidden bad news leads to extreme downside risk, while the managerial short-termism literature explains why managers may take actions that improve short-term performance at the expense of long-term value. However, less is known about how short-term oriented real operating decisions become linked to future crash risk, and under what governance and information conditions this link is weakened or amplified. This study addresses this gap by examining REM-based managerial short-termism as a behavioral source of bad-news accumulation and by analyzing CEO incentive duration and analyst coverage as two complementary boundary conditions.

This issue is closely related to the growing literature on managerial short-termism. Financial markets often respond strongly to short-term performance indicators, and executive compensation is frequently tied to near-term stock price performance (38). Under these conditions, managers face incentives to meet short-term targets. Prior research shows that such incentives are associated with real earnings management and reductions in long-term investment (11–15). While these actions may temporarily improve reported performance, they can also delay the recognition of unfavorable developments.

Motivated by these considerations, the present study examines whether managerial short-termism contributes to firm-level financial instability through its impact on stock price crash risk. By identifying managerial horizon incentives as a behavioral source of stock price crash risk, this study provides evidence on how corporate governance and managerial behavior may influence broader economic stability and socioeconomic conditions relevant to welfare research. Given the multidimensional nature of managerial short-termism, this study focuses on REM as an observable behavioral manifestation that is closely related to short-horizon performance management and delayed recognition of adverse information.

Using real earnings management (REM) as a proxy for managerial short-termism and standard crash-risk measures based on weekly stock returns, including negative conditional skewness (NCSKEW) and down-to-up volatility (DUVOL), we examine whether short-horizon managerial behavior is associated with firm-level crash risk. We further investigate how executive incentive structures shape this relationship. Shorter incentive horizons may increase managers’ sensitivity to current stock prices and strengthen incentives to delay unfavorable disclosures (16), whereas longer incentive horizons may align managerial decisions with long-term firm value. Recent evidence shows that longer CEO equity incentive duration is associated with lower expected crash risk (17).

The health economics relevance of firm-level financial instability should be understood through broader socioeconomic pathways rather than as a directly tested health outcome mechanism. Managerial short-termism may increase firm-level financial instability, which can contribute to socioeconomic stress through employment uncertainty, income volatility, household economic insecurity, and disruptions in essential services. These channels are consistent with the socioeconomic determinants of health framework, which emphasizes that employment conditions, income, access to resources, and healthcare access are important non-medical determinants of health and wellbeing (3, 18, 19). Prior health economics research further shows that economic insecurity is associated with poorer mental health, while firm-level financial frictions can adversely affect employee mental health through workplace and household economic stress (20, 21). In this sense, firm-level crash risk is examined in this study as an intermediate indicator of financial instability, while downstream health-related outcomes are left for future research.

This study contributes to three strands of literature. First, it extends research on stock price crash risk by identifying managerial short-termism as a behavioral source of future downside tail risk. Prior studies have largely emphasized information opacity, disclosure quality, and monitoring mechanisms, whereas this study focuses on short-term oriented managerial behavior as an upstream source of bad-news accumulation. Second, it contributes to the managerial myopia literature by showing that REM-based short-termism is associated not only with operating and reporting distortions, but also with future extreme downside risk in capital markets. Third, it contributes to the corporate governance literature by showing that longer executive incentive horizons and stronger analyst monitoring attenuate the association between managerial short-termism and future crash risk. Beyond these direct contributions, the findings may be relevant to health economics research on socioeconomic determinants of health because firm-level financial instability can affect employment security, income stability, and access to essential services.

The remainder of the study proceeds as follows. Section 2 reviews the related literature and develops the hypotheses. Section 3 describes the data and empirical methodology. Section 4 presents the empirical results, and Section 5 concludes.

## 2 Literature review and hypothesis development

Existing research on stock price crash risk has mainly explained crash events through the delayed disclosure of bad news, information opacity, weak governance, and insufficient external monitoring. This stream of research has clarified how hidden negative information accumulates and is eventually released into the market. However, it gives less attention to the managerial behaviors that may generate or sustain such bad-news accumulation before the crash occurs. At the same time, the managerial short-termism literature shows that managers may manipulate real operating decisions to meet short-term performance targets, but it has less directly examined whether these short-term oriented actions translate into extreme future downside risk. This study connects these two streams by treating REM as an observable manifestation of managerial short-termism and linking REM-based short-termism to future stock price crash risk.

### 2.1 Managerial short-termism and stock price crash risk

Managerial short-termism provides a behavioral explanation for why unfavorable information may accumulate before stock price crashes occur. While the crash-risk literature has emphasized information opacity and weak monitoring, it has paid less attention to how managers’ short-horizon operating decisions may create conditions for delayed recognition of adverse information. The managerial short-termism literature, in turn, explains why managers may prioritize short-term reported performance, but it has less directly examined the implications of such behavior for future extreme downside risk. Building on this gap, this section develops the argument that REM-based managerial short-termism may contribute to future stock price crash risk through the delayed recognition of adverse information.

Managerial short-termism refers to a tendency to prioritize actions that improve short-term performance at the expense of long-term value creation. Theoretical work in agency and contracting frameworks argues that short-horizon incentives induce managers to substitute real operating decisions for value-maximizing actions when near-term performance is heavily weighted in compensation (16, 22, 39). Empirical studies subsequently document this prediction: when managers face strong short-horizon incentives, they are more likely to engage in real activities manipulation, such as accelerating sales, overproducing inventory, or reducing discretionary expenditures in order to meet short-term earnings targets (12, 14, 15). More recent empirical evidence further shows that these incentives are reinforced by disclosure practices and earnings guidance that emphasize short-term results, which may distort firms’ investment and reporting decisions over time (11, 13). Although such actions may temporarily improve reported performance, they can delay the recognition of underlying operational problems.

The bad-news hoarding framework, originally developed as a theoretical model by Jin and Myers (5), predicts that managerial short-termism may contribute to delayed information disclosure. In this framework, when unfavorable developments are not disclosed promptly, stock prices may diverge from fundamental values, and accumulated negative information may eventually be released within a short period, leading to abrupt price adjustments. Subsequent empirical research has tested and supported this prediction: Hutton et al. (4), Kim et al. (9), and Callen and Fang (6) document that firms with weaker information environments and greater managerial discretion over disclosure are more likely to experience severe downside tail events. Complementary empirical work further suggests that managerial characteristics and decision styles, such as CEO age and career background, are associated with differences in stock price crash risk (7, 23). These findings suggest that stock price crash risk is partly related to managerial incentives and disclosure behavior.

Career-related incentives may further strengthen this mechanism. Managers who are evaluated primarily on short-term outcomes face pressure to meet near-term performance targets. Under these conditions, managers may delay the disclosure of unfavorable information and place greater emphasis on short-term indicators of firm performance. Empirical evidence shows that short-horizon incentives affect both managerial behavior and firms’ exposure to risk (13, 14). Evidence on managerial communication also suggests that narrative tone management is associated with future stock price crash risk (24, 37).

Overall, prior studies have established that stock price crash risk is closely related to bad-news hoarding and weak information environments, and that managerial short-termism can distort real operating decisions. What remains less clear is whether short-term oriented real operating behavior serves as a behavioral source of future crash risk. This study addresses this gap by linking REM-based managerial short-termism to the delayed incorporation of adverse information into stock prices. Based on this reasoning, the following hypothesis is proposed:

*Hypothesis 1*. Managerial short-termism is positively associated with future stock price crash risk.

### 2.2 CEO incentive horizon and the short-termism–crash risk relationship

From a theoretical standpoint, agency theory suggests that managerial behavior is shaped not only by the level of compensation incentives but also by the time horizon embedded in incentive contracts (22, 25). Theoretical analyses by Bolton et al. (16) predict that when equity-based compensation is structured over short horizons, managers become more sensitive to current stock price movements and may have stronger incentives to delay unfavorable disclosures in order to protect near-term valuations. Under such conditions, the short-term benefits of concealing bad news may exceed potential long-term costs.

Existing research has shown that longer incentive horizons can reduce managerial myopia and lower firms’ exposure to crash risk. For example, Gu et al. (17) document that longer CEO equity incentive duration is associated with lower expected stock price crash risk, while Wruck and Wu (26) provide related evidence on incentive structure and managerial behavior. However, this literature has paid less attention to whether incentive horizon changes the extent to which REM-based managerial short-termism is translated into future crash risk. This distinction is important because incentive duration may not only affect crash risk directly, but also moderate the consequences of short-term oriented managerial behavior.

When executive incentives emphasize short-term performance, managers may have stronger incentives to delay the disclosure of unfavorable information. In this setting, managerial short-termism is more likely to translate into stock price crash risk. In contrast, longer incentive horizons may reduce the benefits of short-term concealment and align managerial decisions more closely with long-term firm value. Therefore, the effect of managerial short-termism on stock price crash risk is expected to be stronger when executive incentive horizons are shorter.

*Hypothesis 2*. The positive association between managerial short-termism and stock price crash risk is stronger when executive incentive horizons are shorter.

### 2.3 External monitoring and the short-termism–crash risk relationship

If managerial short-termism affects stock price crash risk through delayed information disclosure, stronger external monitoring and a more transparent information environment may mitigate this effect. Information intermediaries such as financial analysts, institutional investors, and disclosure rating agencies can reduce managerial discretion by accelerating the incorporation of firm-specific information into prices and increasing the cost of withholding unfavorable information (36).

Existing research has established that disclosure quality and analyst coverage are closely related to stock price crash risk (8, 10, 27). Recent studies further suggest that analysts’ incentives and career concerns influence how actively they identify and disseminate negative information (28). However, less is known about whether external monitoring weakens the specific link between REM-based managerial short-termism and future crash risk. This distinction is important because analyst coverage may not only reduce crash risk directly, but also limit the extent to which short-term managerial behavior leads to the delayed incorporation of adverse information.

When firms operate in environments with stronger monitoring, opportunities to conceal unfavorable information become more limited (29, 30). As a result, negative information is more likely to be incorporated into prices gradually, reducing the probability of abrupt price adjustments (31–33). Under these conditions, the link between managerial short-termism and stock price crash risk is expected to be weaker. The broader socioeconomic and health economics relevance of firm-level financial instability is discussed in Section 2.4.

*Hypothesis 3*. The positive association between managerial short-termism and stock price crash risk is weaker when external monitoring is stronger.

### 2.4 Socioeconomic and health economics relevance

Although the empirical analysis does not directly test health outcomes, the study may inform health economics research by examining a governance-related source of financial instability that can shape socioeconomic determinants of health. Financial instability at the firm level can affect employment security, income stability, household economic insecurity, and access to essential services. These channels are consistent with the broader public health and health economics literature on socioeconomic conditions and population wellbeing. Accordingly, this study treats health-related implications as broader pathways for future research rather than as directly tested outcomes.

## 3 Methodology

### 3.1 Sample construction and data sources

The sample comprises all non-financial A-share firms listed on the Shanghai and Shenzhen Stock Exchanges over the period 2009–2024. Firm-year observations are obtained from the China Stock Market and Accounting Research (CSMAR) database, which provides comprehensive information on firms’ financial statements, executive characteristics, stock returns, and analyst coverage for Chinese listed companies. Financial firms are excluded because their accounting practices, regulatory environments, and capital structures differ fundamentally from those of non-financial firms, limiting their comparability in studies of stock price crash risk (6, 9).

Firm-level accounting variables, CEO compensation and incentive information, and analyst coverage data are drawn from CSMAR. Weekly stock return data are used to construct firm-level measures of stock price crash risk. Following Hutton et al. (4) and Kim et al. (9), we require a minimum of 30 weekly return observations within each firm-year to ensure reliable estimation of stock price crash risk. Observations with missing values for key variables are excluded. After applying these standard screening procedures, the final baseline sample consists of approximately 2,412 firms and 27,856 firm-year observations.

The baseline, moderation, dynamic, and supplementary analyses are conducted using the same screening criteria to ensure comparability across specifications. For first-difference specifications, the number of observations may be lower because differencing removes the first available observation for each firm. The reported number of observations in each table reflects the actual estimation sample used in that specification.

### 3.2 Variable definitions

#### 3.2.1 Dependent variables: stock price crash risk

Firm-level stock price crash risk is measured using two widely adopted indicators based on firm-specific weekly returns: negative conditional skewness (NCSKEW) and down-to-up volatility (DUVOL).

Following Hutton et al. (4) and Kim et al. (9), we first estimate an expanded market model that includes contemporaneous, lagged, and leading market returns to control for nonsynchronous trading effects (34). The residuals from this model represent firm-specific weekly returns after removing market-wide movements. NCSKEW captures the asymmetry of these residual returns, while DUVOL compares the volatility of negative residual returns with that of positive residual returns. Larger values of either measure indicate a higher degree of stock price crash risk.

Both measures are designed to capture extreme stock price crash risk arising from the concentrated release of accumulated negative information, rather than routine stock price fluctuations (5, 6, 9). All crash-risk variables are constructed in year *t* + 1 relative to explanatory variables measured in year t, which mitigates concerns about reverse causality and is consistent with the delayed realization of hoarded bad news.

To assess the robustness of our findings, we further conduct analyses using alternative specifications of crash-risk measures and model designs, as reported in the robustness section.

#### 3.2.2 Key independent variable: managerial short-termism

Managerial short-termism is a multidimensional construct and cannot be fully captured by any single empirical measure. In this study, we use real earnings management (REM) as the main proxy because it captures one important and observable manifestation of short-horizon managerial behavior: the use of operating decisions to improve current-period performance at the expense of longer-term value.

Following Roychowdhury (15), REM is constructed from abnormal cash flows from operations, abnormal production costs, and abnormal discretionary expenditures. Each abnormal component is standardized within industry-year cells before aggregation. The standardized sum of these abnormal components forms a composite REM index (a unit-free standardized score), with higher values indicating a greater reliance on short-term oriented operational actions. In our sample, REM ranges from −0.60 to 0.86 with a standard deviation of 0.18 (see Table 1).

**Table 1:** Descriptive statistics for all variables.

Variable	Mean	Std.	Min	25%	50%	75%	Max
NCSKEW (*t* + 1)	−0.110	0.197	−0.892	−0.242	−0.110	0.024	0.647
DUVOL (*t* + 1)	−0.109	0.197	−0.878	−0.240	−0.108	0.024	0.676
REM	0.111	0.184	−0.600	−0.013	0.113	0.235	0.858
CEO incentive duration	3.600	1.071	0.500	2.866	3.600	4.327	7.710
Analyst coverage	5.672	2.678	0.000	4.000	5.000	7.000	24.000
Firm size	21.217	0.624	18.459	20.799	21.214	21.641	23.616
Leverage	0.453	0.144	0.020	0.356	0.454	0.552	0.950
ROA	0.040	0.061	−0.241	−0.001	0.039	0.081	0.277
Market-to-book	1.744	0.695	0.200	1.268	1.747	2.214	4.680
Return volatility	0.268	0.061	0.050	0.227	0.268	0.310	0.534
Turnover	0.742	0.355	0.010	0.495	0.739	0.985	2.131
Firm age	15.930	6.199	1.000	11.000	16.000	20.000	31.000

This table presents descriptive statistics for the main variables used in the empirical analysis. Stock price crash risk is measured using negative conditional skewness (NCSKEW) and down-to-up volatility (DUVOL). The sample includes 2,412 firms and 27,856 firm-year observations over the period 2009–2024. Reporting units are as follows: NCSKEW and DUVOL are unit-free skewness/volatility-based measures; REM is a unit-free standardized composite index following Roychowdhury (15); CEO Incentive Duration is reported in years; Analyst Coverage is the number of analysts issuing forecasts in the year; Firm Size is the natural logarithm of total assets; Leverage, ROA, Market-to-Book, Return Volatility, and Turnover are reported as ratios; Firm Age is reported in years.

Our use of REM is motivated by both theory and research design. First, prior studies link managerial myopia and short-horizon incentives to real operating distortions rather than purely accrual-based reporting choices (12–14). Second, REM is particularly suitable for the present study because the proposed mechanism runs from short-term managerial behavior to future stock price crash risk through delayed recognition of adverse information. Real operating actions can temporarily mask underlying performance deterioration and are often less transparent to outside investors in real time. For this reason, REM provides a behavior-based proxy that is closely aligned with the bad-news hoarding mechanism examined in this paper.

At the same time, we do not interpret REM as a complete measure of managerial short-termism. Rather, it should be viewed as one observable and theory-consistent proxy for a broader construct. Alternative measures, such as compensation-based indicators, disclosure-related proxies, or textual measures, may capture different dimensions of managerial horizon and provide complementary evidence.

While REM captures short-horizon operating behavior, it may also reflect other firm-level conditions. For example, firms experiencing operational stress or financing constraints may adjust real activities in ways that resemble earnings management. In addition, industry-level competition and demand fluctuations may influence production and sales decisions. Therefore, REM should be interpreted as a proxy that captures short-term oriented behavior with potential noise from other economic factors.

Despite these considerations, REM remains suitable in this study because it captures operating actions that can temporarily mask underlying performance and delay the incorporation of adverse information into stock prices, which is directly aligned with the bad-news hoarding mechanism underlying stock price crash risk. Therefore, the REM measure should be interpreted as an informative but potentially noisy proxy for managerial short-termism.

#### 3.2.3 Moderating variables

Two moderating variables are introduced to examine how internal incentive structures and external monitoring conditions shape the relationship between managerial short-termism and stock price crash risk.

The first moderator captures the executive incentive horizon. Following Gu et al. (17), we use CEO equity incentive duration, measured in years, to measure the effective time horizon of equity-based compensation. This variable reflects the remaining duration of CEO equity incentives (in years) and captures the effective planning horizon embedded in executive compensation. In our sample, this variable ranges from 0.50 to 7.71 years (see Table 1). A longer incentive duration implies a longer planning horizon and weaker pressure to focus on short-term stock price performance. Shorter incentive duration, in contrast, increases managers’ sensitivity to near-term price movements and strengthens incentives to postpone unfavorable disclosures.

The second moderator captures the strength of external monitoring and the information environment. We use analyst coverage, measured as integer count of distinct financial analysts issuing earnings forecasts for a firm in a given year (unit: number of analysts). Analyst coverage reflects the intensity of information production and external scrutiny faced by a firm. Higher coverage implies more frequent information processing, faster incorporation of firm-specific news into prices, and higher reputational costs of withholding negative information. Prior studies show that firms followed by more analysts tend to exhibit lower stock price crash risk and stronger discipline on managerial behavior (8, 10, 27).

Analyst coverage enters the empirical models both as a main effect and as an interaction term with managerial short-termism. This specification allows us to examine whether external monitoring weakens the extent to which short-term managerial behavior translates into stock price crash risk. In addition, recent evidence shows that analysts’ own incentives and career concerns influence their willingness to reveal unfavorable information, which further affects the effectiveness of monitoring (28).

To examine whether the association is stronger in sectors where firm-level instability may have broader socioeconomic relevance, we construct an indicator for essential-service industries. These industries include healthcare services, pharmaceuticals, food supply, and transportation. Their operations are more closely related to employment stability, supply continuity, and access to essential goods and services. Therefore, disruptions in these sectors may have stronger socioeconomic implications. The detailed classification is reported in Table A9 in the Appendix.

### 3.3 Control variables

A set of standard firm-level controls is included to account for factors that may influence stock price crash risk. These variables include firm size (the natural logarithm of total assets), leverage (total liabilities divided by total assets), profitability (return on assets, ROA), market-to-book ratio, stock return volatility, trading turnover, firm age, and analyst coverage.

Firm size controls for differences in information environments and investor attention. Leverage reflects financial risk and incentives to conceal adverse information. Profitability and market-to-book ratio control for operating performance and growth opportunities, which affect both disclosure behavior and stock price dynamics. Stock return volatility and trading turnover proxy for the inherent riskiness and liquidity of a firm’s stock. Firm age reflects differences in organizational maturity and disclosure practices between younger and more established firms. Analyst coverage is included to control for baseline differences in information production and monitoring intensity across firms, independent of its moderating role examined in the interaction analysis.

All control variables are measured in year t and used to explain stock price crash risk in year *t* + 1, which is consistent with the intertemporal nature of information concealment and risk realization. All continuous variables are winsorized at the 1st and 99th percentiles to limit the influence of extreme observations.

All baseline, dynamic, and moderation regressions include firm and year fixed effects, and standard errors are clustered at the firm level to account for within-firm serial correlation. In the first-difference specification, firm fixed effects are not included because differencing removes time-invariant firm-specific effects; year fixed effects are retained to absorb common time-varying shocks.

### 3.4 Empirical model specifications

#### 3.4.1 Baseline model

Following the standard approach in the stock price crash risk literature (6, 9), we begin with a baseline fixed-effects specification to examine whether managerial short-termism predicts future stock price crash risk:

$$Crash_{i,t+1}=\alpha +\beta_{1}REM_{i,t}+\gamma X_{i,t}+\mu_{i}+\lambda_{t}+\varepsilon_{i,t}$$ (1)

In Equation 1, $Crash_{i,t+1}$ denotes firm-level stock price crash risk measured in year *t* + 1, proxied by negative conditional skewness (NCSKEW) and down-to-up volatility (DUVOL), both constructed from firm-specific weekly stock returns. The key explanatory variable, ${\mathrm{REM}}_{i,t}$ , is the real earnings management index for firm *i* in year *t*, constructed following Roychowdhury (15) and widely used as a proxy for managerial short-termism (12–14). Higher values of ${\mathrm{REM}}_{i,t}$ indicate a stronger tendency for managers to prioritize short-horizon performance through real operational actions.

The coefficient $\beta_{1}$ captures the effect of managerial short-termism on future stock price crash risk. The vector $X_{i,t}$ includes a standard set of control variables shown to affect stock price crash risk in prior studies, including firm size, leverage, profitability, market-to-book ratio, stock return volatility, trading turnover, firm age, and analyst coverage. Firm fixed effects $\mu_{i}$ control for time-invariant unobserved heterogeneity, while year fixed effects $\lambda_{t}$ absorb common macroeconomic shocks. Standard errors are clustered at the firm level to account for within-firm serial correlation.

To provide supplementary evidence, we employ an instrumental variable approach using the industry-year average level of real earnings management (REM), excluding the focal firm, as an instrument for firm-level REM.

The economic rationale for this instrument is that firms operating within the same industry are subject to similar competitive pressures, production environments, and performance benchmarks, which influence their incentives to engage in short-term oriented operating decisions. This shared environment generates cross-sectional variation in REM at the industry level, satisfying the relevance condition.

The exclusion restriction requires that industry-level REM affects firm-level stock price crash risk only through its influence on the firm’s own operating behavior. While industry-wide conditions may affect overall risk exposure, the use of the industry average excluding the focal firm reduces the likelihood that the instrument directly captures firm-specific stock price crash risk.

Empirically, the first-stage results indicate that the instrument is positively and statistically significantly associated with firm-level REM. The corresponding first-stage *F*-statistic is 66.24 (see Appendix Table A6), which exceeds the conventional threshold of 10 and confirms strong instrument relevance.

At the same time, we acknowledge that the exclusion restriction may not hold perfectly if industry-level shocks simultaneously affect both operating behavior and stock price crash risk. Therefore, the instrumental variable estimates should be interpreted as providing supportive rather than definitive causal evidence.

The first-stage *F*-statistic should therefore be interpreted only as evidence of instrument relevance and not as evidence that the exclusion restriction holds.

#### 3.4.2 Dynamic specification

Stock price crash risk may exhibit persistence over time, reflecting firms’ underlying disclosure practices, governance culture, or investor perceptions. To account for this possibility and to ensure that the estimated effect of managerial short-termism is not driven by serial dependence in stock price crash risk, we estimate the following dynamic specification, as specified in Equation (2):

$$Crash_{i,t}=\alpha +\beta_{1}REM_{i,t}+\beta_{2}Crask_{i,t}+\gamma X_{i,t}+\mu_{i}+\lambda_{t}+\varepsilon_{i,t}$$ (2)

By including the lagged dependent variable, $Crash_{i,t+1}$ , this model controls for persistence in stock price crash risk. The coefficient on $REM_{i,t}$ therefore reflects the incremental effect of managerial short-termism after accounting for firms’ historical exposure to stock price crash risk. This specification is used to verify that the main results are not driven by simple persistence in stock price crash risk or by autocorrelation in return skewness (10).

The lagged crash-risk variable is introduced only in the dynamic specification and is therefore not included in the descriptive statistics or correlation matrix, which focus on baseline explanatory variables. All regressions include firm and year fixed effects.

While the inclusion of a lagged dependent variable in a fixed-effects panel may introduce bias (40), particularly in panels with limited time dimensions, the relatively long time dimension of the sample period in the present study helps mitigate this concern. Moreover, the dynamic specification is not intended as the primary identification strategy but rather as a robustness check to account for persistence in stock price crash risk.

Alternative estimators designed for dynamic panels, for example, system GMM, may further address this issue. However, given the potential sensitivity of such estimators to instrument proliferation and finite-sample properties, we retain the fixed-effects specification. Accordingly, the dynamic results are interpreted as supplementary robustness evidence rather than definitive causal estimates.

#### 3.4.3 Moderation models

To examine whether the effect of managerial short-termism on stock price crash risk depends on executive incentive horizons and external monitoring conditions, we extend the baseline model by introducing interaction terms, as shown in Equation (3):

$$\begin{array}{l} Crash_{i,t+1}=\alpha +\beta_{1}REM_{i,t}+\beta_{2}Z_{i,t}+\beta_{3}\left(REM_{i,t}\times Z_{i,t}\right)\\ +\gamma X_{i,t}+FE+\varepsilon_{i,t} \end{array}$$ (3)

where $Z_{i,t}$ represents moderating variables that capture either internal incentive horizons or external monitoring strength. Specifically, executive incentive horizon is measured by CEO equity incentive duration following Gu et al. (17), while external monitoring is proxied by analyst coverage.

The interaction term $REM_{i,t}\times Z_{i,t}$ allows us to test whether the impact of managerial short-termism on stock price crash risk is amplified or attenuated by managerial incentives and information environments. In the incentive-horizon regressions, a significantly positive $\beta_{3}$ indicates that shorter incentive horizons strengthen the effect of short-termism on stock price crash risk. In the monitoring regressions, a significantly negative $\beta_{3}$ implies that stronger external monitoring mitigates the transmission of managerial short-termism into stock price crash risk (14, 17).

When analyst coverage serves as the moderating variable, it is included both as a main effect and as an interaction term with REM. In these specifications, analyst coverage is not double-counted as a separate control variable.

#### 3.4.4 Change-on-change specification

To further address concerns related to time-invariant omitted variables and unobserved firm-specific risk culture, we estimate a first-difference model following the change-on-change approach used by Ladika (14), as specified in Equation (4):

$$\Delta CrashRisk_{i,t+1}=\alpha +\beta_{1}\Delta REM_{i,t}+\gamma^{\prime }\Delta X_{i,t}+\lambda_{t}+\varepsilon_{i,t}$$ (4)

where Δ denotes the first difference operator. $�CrashRisk_{i,t+1}$ denotes the first difference in firm-level stock price crash risk, measured using NCSKEW or DUVOL. $�REM_{i,t}$ captures managerial short-termism, and $�X_{i,t}$ represents the vector of control variables. This specification focuses on whether within-firm changes in managerial short-termism are associated with subsequent changes in stock price crash risk. By differencing the data, the model removes time-invariant firm-specific characteristics, including stable governance culture, persistent disclosure style, and other fixed firm attributes. Therefore, firm fixed effects are not included in this specification. Year fixed effects $\lambda_{t}$ , are retained to control for common time-varying shocks, such as macroeconomic conditions, regulatory changes, and market-wide fluctuations that may affect changes in stock price crash risk across firms. Standard errors are clustered at the firm level.

## 4 Empirical result

### 4.1 Descriptive statistics

Table 1 presents descriptive statistics for the variables used in the empirical analysis over the period 2009–2024.

The proxy for managerial short-termism, real earnings management (REM), exhibits substantial variation across firms, with a mean of 0.111 and a standard deviation of 0.184. This magnitude is broadly consistent with the range documented in prior studies using Chinese listed firms, suggesting that the observed level of real earnings management in our sample is not driven by a small number of extreme observations.

The two measures of stock price crash risk, NCSKEW and DUVOL, also display meaningful dispersion. The distribution of NCSKEW ranges from −0.892 to 0.647, indicating that a subset of firms experiences relatively extreme negative return realizations. This variation is consistent with the interpretation of stock price crash risk as a tail event that occurs infrequently but with large magnitude. Similar dispersion is observed for DUVOL, suggesting that stock price crash risk varies meaningfully across firms.

With respect to the moderating variables, both CEO incentive duration and analyst coverage exhibit sufficient variation to support the fixed-effects design. CEO incentive duration ranges from 0.5 to 7.71 years, indicating that executive planning horizons differ substantially both across firms and over time. Analyst coverage ranges from 0 to 24, reflecting considerable differences in external monitoring intensity. Importantly, both variables display variation within firms over time, which is necessary for identification in a fixed-effects framework.

These patterns are broadly consistent with findings documented in prior empirical studies on stock price crash risk and managerial behavior in Chinese capital markets. Taken together, the descriptive statistics suggest that the key variables exhibit sufficient cross-sectional and within-firm variation to support the empirical analysis.

Table 2 presents the pairwise Pearson correlations among the main variables. REM is positively correlated with both measures of stock price crash risk (NCSKEW and DUVOL), suggesting that firms with greater real earnings management tend to exhibit higher stock price crash risk. Although the correlation between NCSKEW and DUVOL is moderate, the two measures capture different dimensions of stock price crash risk and are therefore not expected to be identical.

**Table 2:** Pearson correlation matrix.

Variables	(1)	(2)	(3)	(4)	(5)	(6)	(7)	(8)	(9)	(10)	(11)	(12)
(1) NCSKEW	1.000											
(2) DUVOL	0.214	1.000										
(3) REM	0.071	0.063	1.000									
(4) CEO incentive duration	−0.193	−0.165	−0.121	1.000								
(5) Analyst coverage	−0.025	−0.031	−0.034	0.214	1.000							
(6) Firm size	0.216	0.198	0.338	−0.174	0.655	1.000						
(7) Leverage	0.191	0.176	0.083	0.097	0.161	0.239	1.000					
(8) ROA	−0.156	−0.142	−0.074	−0.068	−0.124	−0.198	−0.173	1.000				
(9) Market-to-book	−0.004	−0.006	0.009	0.011	0.001	−0.000	0.009	0.008	1.000			
(10) Return volatility	0.033	0.028	0.001	0.002	0.003	0.000	−0.006	−0.009	−0.003	1.000		
(11) Turnover	0.007	0.006	0.009	0.010	0.008	0.010	0.017	−0.004	0.082	0.004	1.000	
(12) Firm age	0.032	0.029	0.010	0.012	−0.001	0.002	0.013	−0.002	0.060	−0.028	0.080	1.000

This table presents Pearson correlation coefficients among the main variables used in the analysis. Regression results using DUVOL are reported in the robustness tests. The lower triangular matrix is reported for brevity. Variable definitions follow Section 3.2.

Consistent with prior studies, stock price crash risk is positively correlated with return volatility and leverage. Analyst coverage is negatively correlated with stock price crash risk, indicating that firms subject to stronger external monitoring tend to experience lower stock price crash risk. Overall, the pairwise correlations among the explanatory variables are relatively small to moderate, suggesting that multicollinearity is unlikely to be a concern in the subsequent regression analyses.

Table 3 reports the baseline fixed-effects and dynamic regression results examining the relationship between managerial short-termism and future stock price crash risk. Across both specifications, the coefficient on real earnings management (REM) is positive and statistically significant, indicating that firms engaging more intensively in short-term oriented operational actions face a higher likelihood of extreme downside stock price movements in the subsequent year.

**Table 3:** Baseline model and dynamic specification (NCSKEW).

Variables	Baseline model	Dynamic specification
REM	0.184^***^ (4.21)	0.137^***^ (3.46)
Lagged stock price crash risk		0.293^***^ (6.17)
Firm size	−0.021^**^ (−2.34)	−0.018^**^ (−2.11)
Leverage	0.067^***^ (3.01)	0.055^**^ (2.45)
Return on assets	−0.142^***^ (−4.07)	−0.119^***^ (−3.58)
Firm fixed effects	Yes	Yes
Year fixed effects	Yes	Yes
Adjusted *R*^2^	0.261	0.338
Observations	27,856	27,856

The dependent variable is firm-level stock price crash risk measured by negative conditional skewness (NCSKEW) in year *t* + 1. Managerial short-termism is proxied by real earnings management (REM) measured in year *t*. The baseline model estimates the association between REM and future stock price crash risk controlling for firm and year fixed effects. The dynamic specification additionally includes lagged stock price crash risk to account for persistence in stock price crash risk. Control variables are fully reported in Appendix Table A1. All models include firm and year fixed effects. Standard errors are clustered at the firm level. *t*-statistics are reported in parentheses. ^***^, ^**^, and ^*^ denote statistical significance at the 1, 5, and 10% levels, respectively. Variable units follow Table 1: REM is a unit-free standardized composite index; CEO Incentive Duration is in years; Analyst Coverage is in number of analysts.

The estimated coefficients indicate that higher levels of REM are associated with greater stock price crash risk, consistent with the view that real earnings management facilitates the temporary concealment of adverse information and increases the probability of a clustered release of bad news.

Importantly, the positive effect of REM remains statistically significant after controlling for lagged stock price crash risk in the dynamic specification, suggesting that the baseline results are not driven by persistence in stock price crash risk or simple autocorrelation in return skewness. Overall, the evidence in Table 3 provides support for Hypothesis 1, documenting a systematic link between managerial short-termism and future stock price crash risk.

For transparency, full regression results including all control variables are reported in Appendix Table A1. The complete moderation specifications are presented in Appendix Table A2.

Table 4 reports the moderation results examining whether executive incentive horizons shape the relationship between managerial short-termism and future stock price crash risk. The coefficient on real earnings management (REM) remains positive and statistically significant, indicating that short-term oriented managerial behavior is associated with higher stock price crash risk.

**Table 4:** Moderation: CEO equity incentive duration (NCSKEW).

Variables	Moderation model
REM	0.203^***^ (4.56)
Duration	−0.041^***^ (−3.72)
REM × Duration	−0.028^**^ (−2.19)
Firm size	−0.019^**^ (−2.08)
Leverage	0.061^**^ (2.48)
Return on assets	−0.131^***^ (−3.91)
Firm fixed effects	Yes
Year fixed effects	Yes
Adjusted *R*^2^	0.297
Observations	27,856

The dependent variable is firm-level stock price crash risk measured by negative conditional skewness (NCSKEW) in year *t* + 1. Managerial short-termism is proxied by real earnings management (REM) measured in year *t*. Duration refers to CEO equity incentive duration (measured in years) and captures the effective horizon of executive compensation. The interaction term (REM × Duration) tests whether longer incentive horizons mitigate the impact of managerial short-termism on future stock price crash risk. Variable units follow Table 1: REM is a unit-free standardized composite index; CEO Incentive Duration is in years; Analyst Coverage is in number of analysts.

More importantly, the interaction term between REM and Duration is negative and statistically significant, indicating that longer incentive horizons attenuate the positive association between managerial short-termism and stock price crash risk.

To illustrate the economic magnitude of the moderation effects, we evaluate the implied change in stock price crash risk when REM increases from the 25th percentile to the 75th percentile. Based on Table 1, this interquartile change in REM is 0.248. Using the estimates in Table 4, the implied increase in NCSKEW is approximately 0.031 when CEO incentive duration is at its 25th percentile, but only about 0.020 when duration is at its 75th percentile. This indicates that longer incentive horizons reduce the effect of managerial short-termism on stock price crash risk by roughly 34%.

This result suggests that shorter incentive horizons strengthen managers’ incentives to delay the disclosure of unfavorable information, whereas longer incentive horizons align managerial incentives with long-term firm value and reduce the accumulation of hidden negative information.

Overall, the results in Table 4 provide support for Hypothesis 2 and indicate that executive incentive horizon acts as an important boundary condition in the relationship between managerial short-termism and stock price crash risk.

Table 5 reports the moderation results examining whether external monitoring conditions shape the relationship between managerial short-termism and future stock price crash risk. The coefficient on real earnings management (REM) remains positive and statistically significant, indicating that short-term oriented managerial behavior is associated with higher stock price crash risk.

**Table 5:** Moderation analysis: analyst coverage and stock price crash risk (NCSKEW).

Variables	Moderation model
REM	0.176^***^ (3.98)
Analyst coverage	−0.014^**^ (−2.21)
REM × Analyst coverage	−0.009^*^ (−1.78)
Firm size	−0.017^*^ (−1.86)
Leverage	0.058^**^ (2.33)
Firm fixed effects	Yes
Year fixed effects	Yes
Adjusted *R*^2^	0.281
Observations	27,856

The dependent variable is stock price crash risk measured by NCSKEW in year *t* + 1. Managerial short-termism is proxied by real earnings management (REM). Analyst coverage measures the intensity of external monitoring. The interaction term examines whether stronger analyst monitoring weakens the effect of managerial short-termism on stock price crash risk. All control variables used in the baseline specification are included in the regression. All models include firm and year fixed effects. *t*-statistics are reported in parentheses. ^***^, ^**^, and ^*^ denote statistical significance at the 1, 5, and 10% levels, respectively. Variable units follow Table 1: REM is a unit-free standardized composite index; CEO Incentive Duration is in years; Analyst Coverage is in number of analysts.

Analyst coverage enters the regression with a significantly negative coefficient, suggesting that firms subject to stronger external monitoring exhibit lower stock price crash risk. More importantly, the interaction term (REM × Analyst coverage) is negative and statistically significant, indicating that stronger analyst monitoring attenuates the positive association between managerial short-termism and stock price crash risk.

Using the estimates in Table 5, the implied increase in NCSKEW associated with an interquartile increase in REM is approximately 0.035 when analyst coverage is at its 25th percentile and about 0.028 when analyst coverage is at its 75th percentile. This pattern is consistent with the negative interaction term and suggests that stronger analyst monitoring attenuates the effect of REM on stock price crash risk. To facilitate interpretation of the interaction effects, marginal-effect illustrations are provided in Appendix Figures A1 and A2.

These findings suggest that the relationship between managerial short-termism and future stock price crash risk varies across firms depending on the surrounding information environment. When external monitoring is stronger, unfavorable information is more likely to be incorporated into the information environment in a timely manner, thereby reducing the accumulation of hidden stock price crash risk.

Overall, the results in Table 5 provide support for Hypothesis 3 and indicate that external monitoring conditions shape the relationship between managerial short-termism and stock price crash risk.

To further assess the robustness of the baseline and moderation results, we conduct additional tests using an alternative measure of stock price crash risk. Specifically, we replace negative conditional skewness (NCSKEW) with down-to-up volatility (DUVOL), which captures stock price crash risk from a volatility-based perspective and complements skewness-based measures.

Table 6 reports the baseline fixed-effects and dynamic regression results using DUVOL as an alternative measure of stock price crash risk. The lagged DUVOL term in the dynamic specification is positive and significant, confirming persistence in stock price crash risk over time. Importantly, the estimated effect of REM remains statistically significant after controlling for this persistence, indicating that the main results are robust when stock price crash risk is measured using an alternative volatility-based metric.

**Table 6:** Baseline model and dynamic specification (DUVOL).

Variables	Baseline model	Dynamic specification
REM	0.159^***^ (3.77)	0.122^***^ (3.01)
Lagged stock price crash risk		0.271^***^ (5.44)
Firm size	−0.018^*^ (−1.92)	−0.015^*^ (−1.78)
Leverage	0.052^**^ (2.29)	0.046^**^ (2.07)
Firm fixed effects	Yes	Yes
Year fixed effects	Yes	Yes
Adjusted *R*^2^	0.214	0.289
Observations	27,856	27,856

The dependent variable is stock price crash risk measured by down-to-up volatility (DUVOL) in year *t* + 1. Managerial short-termism is proxied by real earnings management (REM). REM is measured in year t. All models include firm and year fixed effects. *t*-statistics clustered at the firm level are reported in parentheses. ^***^, ^**^, and ^*^ denote statistical significance at the 1, 5, and 10% levels, respectively. Variable units follow Table 1: REM is a unit-free standardized composite index; CEO Incentive Duration is in years; Analyst Coverage is in number of analysts.

The corresponding moderation results based on CEO equity incentive duration are presented in Table 7. Change-on-change specifications are reported in Appendix Tables A3–A5, which provide consistent supplementary evidence based on within-firm changes over time and alternative crash-risk proxies. Appendix Table A6 reports a supplementary instrumental-variable analysis using industry-year average REM, excluding the focal firm, as an instrument. The first-stage *F*-statistic indicates strong instrument relevance. However, because industry-year conditions may affect crash risk through channels other than the focal firm’s own REM, the exclusion restriction cannot be fully verified. Therefore, these IV estimates are interpreted as supportive robustness evidence rather than as definitive causal evidence.

**Table 7:** Moderation: CEO incentive duration (DUVOL).

Variables	Moderation model
REM	0.171^***^ (3.94)
Duration	−0.037^***^ (−3.21)
REM × Duration	−0.024^**^ (−2.02)
Firm fixed effects	Yes
Year fixed effects	Yes
Adjusted *R*^2^	0.243
Observations	27,856

The dependent variable is stock price crash risk measured by DUVOL in year *t* + 1. Managerial short-termism is proxied by real earnings management (REM). CEO equity incentive duration captures the effective horizon of executive compensation (Duration). The interaction term tests whether longer incentive horizons mitigate the effect of managerial short-termism on stock price crash risk. All models include firm and year fixed effects. *t*-statistics are reported in parentheses. ^***^, ^**^, and ^*^ denote statistical significance at the 1, 5, and 10% levels, respectively. Variable units follow Table 1: REM is a unit-free standardized composite index; CEO Incentive Duration is in years; Analyst Coverage is in number of analysts.

Importantly, the estimated effect of REM remains statistically significant after controlling for crash-risk persistence, indicating that the main results remain robust when stock price crash risk is measured using an alternative volatility-based metric.

Heterogeneity analyses based on corporate governance quality and essential-service industries are reported in Appendix Tables A7 and A8. The results indicate that the association between managerial short-termism and stock price crash risk is more pronounced in sectors where firm-level instability may have broader socioeconomic relevance. This finding suggests that the economic consequences of firm-level financial instability may be more salient in industries related to employment stability, supply continuity, and access to essential goods and services. These results are consistent with the broader argument that corporate financial instability may be relevant to socioeconomic determinants of health, although health outcomes are not directly measured in this study.

Although the additional heterogeneity analyses focus on governance quality and essential-service industries, future research may further examine whether the short-termism crash relationship varies across firm size, ownership structure, competitive intensity, or regional institutional environments.

Table 7 examines whether the moderating role of executive incentive horizons remains robust when stock price crash risk is measured using DUVOL. The coefficient on real earnings management (REM) remains positive and statistically significant, while Duration is negatively associated with stock price crash risk.

More importantly, the interaction term (REM × Duration) is negative and statistically significant, indicating that longer incentive horizons mitigate the positive association between managerial short-termism and stock price crash risk.

This finding mirrors the moderation pattern observed in Table 4 and suggests that executive incentive horizons act as an important boundary condition in the relationship between managerial short-termism and stock price crash risk when stock price crash risk is measured using an alternative proxy (DUVOL).

Overall, the empirical results provide consistent evidence that managerial short-termism is associated with firm-level stock price crash risk. Across baseline, dynamic, and moderation specifications, firms engaging more intensively in real earnings management exhibit a higher likelihood of extreme negative stock price movements.

The moderation analyses further show that this relationship depends on both internal and external governance conditions. Longer executive incentive horizons attenuate the impact of managerial short-termism on stock price crash risk, while stronger external monitoring through analyst coverage also weakens this association. These findings suggest that managerial incentives and information environments jointly influence the extent to which short-term oriented behavior translates into extreme downside outcomes.

Additional robustness tests, including alternative crash-risk measures, change-on-change regressions, supplementary instrumental-variable analysis, and heterogeneity analyses, provide results that are consistent with the baseline findings. However, these analyses should not be interpreted as fully eliminating all endogeneity concerns. The evidence therefore supports a robust association between managerial short-termism and future stock price crash risk, while the causal interpretation should remain cautious.

## 5 Conclusion and discussion

### 5.1 Conclusion

This study investigates the role of managerial short-termism in shaping firm-level stock price crash risk and its implications for financial instability. Using a comprehensive sample of Chinese A-share firms from 2009 to 2024, we document a statistically significant positive association between real earnings management, our primary proxy for managerial short-termism, and subsequent stock price crash risk. This relationship remains robust across alternative crash-risk measures, dynamic specifications accounting for persistence in stock price crash risk, change-on-change regressions exploiting within-firm variation, and supplementary robustness analyses.

Despite these identification strategies, endogeneity concerns cannot be fully ruled out. In particular, reverse causality may arise if firms with higher expected stock price crash risk adjust managerial behavior toward short-term performance management. Although lagged explanatory variables, dynamic specifications, change-on-change regressions, and supplementary IV analysis help reduce some concerns, the exclusion restriction of the instrument may not hold if industry-level conditions simultaneously affect both operating behavior and stock price crash risk. Accordingly, the results should be interpreted as evidence consistent with the proposed behavioral mechanism, rather than definitive causal proof.

Beyond the baseline effect, the analysis shows that the consequences of managerial short-termism are not uniform across firms. Longer executive incentive horizons and stronger external monitoring significantly attenuate the short-termism crash relationship, indicating that incentive alignment and information transparency limit the accumulation of undisclosed adverse information. These findings suggest that stock price crashes can emerge as an endogenous outcome of managerial horizon incentives. By highlighting the governance origins of extreme stock price crash risk, the study provides new evidence on how institutional mechanisms shape firm-level stock price crash risk and financial instability. These findings contribute to the literature by identifying managerial horizon incentives as an important behavioral determinant of stock price crash risk.

Although the present study does not directly examine health outcomes, the findings may be relevant for the broader health economics literature on economic insecurity and social determinants of health. Prior studies suggest that firm-level financial instability can affect employment stability, household income expectations, and access to essential services, which may subsequently influence health-related outcomes. Future research may extend this framework by incorporating regional labor-market indicators, healthcare access measures, or population-level health data to examine these channels more directly.

Rather than providing direct evidence on public health outcomes, this study identifies a governance-related source of firm-level financial instability that may be relevant to broader research on economic insecurity, social determinants of health, and welfare. Therefore, the health economics relevance of the study should be interpreted through socioeconomic pathways rather than through directly tested health outcomes.

### 5.2 Discussion

This study is relevant to the health economics literature through its focus on firm-level financial instability as an upstream economic risk factor. Prior research in health economics has shown that economic shocks, labor-market insecurity, and income volatility are associated with adverse health-related outcomes, including psychological stress, reduced healthcare utilization, and lower household welfare. Within this broader framework, the present study identifies managerial short-termism as a behavioral factor associated with future stock price crash risk. However, the paper does not directly estimate health outcomes. Therefore, the public health implications discussed in this study should be interpreted as being inferred through socioeconomic pathways, including employment instability, income volatility, and economic insecurity.

For corporate boards and compensation committees, the findings highlight the potential risk consequences of short-horizon incentive schemes. Although performance-based compensation is intended to align managerial actions with shareholder interests, excessively short evaluation horizons may encourage the concealment of unfavorable information and increase stock price crash risk. The mitigating role of longer incentive horizons documented in this study suggests that extending vesting periods and emphasizing long-term performance metrics can improve corporate governance and contribute to greater market stability.

From a broader policy perspective, the findings suggest that firm-level financial instability may have wider socioeconomic implications through channels such as employment uncertainty and income volatility. However, the empirical analysis in this study focuses on financial outcomes rather than directly measuring health-related variables.

A related limitation is that managerial short-termism is measured using real earnings management (REM), which captures short-horizon operating behavior but may also reflect other firm-level conditions, such as operational stress or financing constraints. As a result, the measure is subject to potential noise and should be interpreted with caution.

In addition, managerial short-termism is inherently multidimensional and may not be fully captured by a single observable proxy. While REM reflects realized short-horizon operating actions closely related to the mechanism examined in this study, alternative measures such as compensation-based horizon indicators, earnings-guidance pressure, or textual measures of managerial myopia may capture complementary dimensions of short-term behavior. Future research may compare these alternative proxies within a unified framework.

Importantly, the analysis does not establish a direct causal relationship between firm-level financial instability and health outcomes. Instead, it provides evidence consistent with an upstream mechanism that may contribute to broader socioeconomic conditions documented in the health economics literature. Similarly, although the empirical evidence is consistent with a behavioral short-termism channel, the results should not be interpreted as definitive causal evidence linking managerial short-termism to future stock price crash risk.

In addition, the sample is limited to non-financial Chinese A-share firms. Differences in institutional environments, governance systems, disclosure practices, and financial market structures may affect the generalizability of the findings to other economies or financial sectors.

A key limitation of this study is that it does not directly estimate firm-level or population-level health outcomes. Such data are not available in a way that can be consistently linked to listed firms in the present setting. Therefore, the study should not be interpreted as providing direct evidence on health outcomes. Instead, its health economics relevance lies in identifying a governance-related source of firm-level financial instability that may shape socioeconomic conditions, such as employment security, income stability, and access to essential services. Future research may examine whether similar relationships exist in alternative institutional settings and further investigate these socioeconomic transmission channels by linking firm-level financial instability to regional labor-market indicators, healthcare access measures, or population-level health outcomes.

## Data Availability

The original contributions presented in the study are included in the article/Supplementary material, further inquiries can be directed to the corresponding author.

## References

[1] Catalano R Goldman-Mellor S Saxton K Margerison-Zilko C Subbaraman M LeWinn K . The health effects of economic decline. Annu Rev Public Health. (2011) 32:431–50. doi: 10.1146/annurev-publhealth-031210-101146, 21054175 PMC3855327

[2] Ruhm CJ. Are recessions good for your health? Q J Econ. (2000) 115:617–50. doi: 10.1162/003355300554872, 22462190

[3] Stuckler D Basu S. The Body Economic: Why Austerity Kills. New York: Basic Books (2013).

[4] Hutton AP Marcus AJ Tehranian H. Opaque financial reports, R^2^, and crash risk. J Financ Econ. (2009) 94:67–86. doi: 10.1016/j.jfineco.2008.10.003

[5] Jin L Myers SC. R^2^ around the world: new theory and new tests. J Financ Econ. (2006) 79:257–92. doi: 10.1016/j.jfineco.2004.11.003

[6] Callen JL Fang X. Religion and stock price crash risk. J Financ Quant Anal. (2015) 50:169–95. doi: 10.1017/S0022109015000046

[7] Fang X Girardone C Li Y Zeng Y. Generalist CEOs and stock price crash risk. J Bus Financ Acc. (2025) 52:182–221. doi: 10.1111/jbfa.12804

[8] Ho KC Karathanasopoulos A Lo CC Shen X. Information disclosure ratings and stock price crash risk. Rev Quant Finan Acc. (2024) 63:1323–48. doi: 10.1007/s11156-024-01305-0

[9] Kim J-B Li Y Zhang L. Corporate tax avoidance and stock price crash risk: firm-level analysis. J Financ Econ. (2011) 100:639–62. doi: 10.1016/j.jfineco.2010.07.007

[10] Kim J-B Li Y Zhang L. CFOs versus CEOs: equity incentives and crashes. J Financ Econ. (2011) 101:713–30. doi: 10.1016/j.jfineco.2011.03.013

[11] Aghamolla C Hashimoto T. Managerial myopia, earnings guidance, and investment. Contemp Account Res. (2023) 40:166–95. doi: 10.1111/1911-3846.12820

[12] Graham JR Harvey CR Rajgopal S. The economic implications of corporate financial reporting. J Account Econ. (2005) 40:3–73. doi: 10.1016/j.jacceco.2005.01.002

[13] Jo EH Lee JW. Economic policy uncertainty and managerial short-termism. Int Rev Financ Anal. (2024) 93:103216. doi: 10.1016/j.irfa.2024.103216

[14] Ladika T Sautner Z. Managerial short-termism and investment: evidence from accelerated vesting. Rev Financ. (2020) 24:305–44. doi: 10.1093/rof/rfz012

[15] Roychowdhury S. Earnings management through real activities manipulation. J Account Econ. (2006) 42:335–70. doi: 10.1016/j.jacceco.2006.01.002

[16] Bolton P Scheinkman J Xiong W. Executive compensation and short-termist behaviour in speculative markets. Rev Econ Stud. (2006) 73:577–610. doi: 10.1111/j.1467-937X.2006.00388.x

[17] Gu Z Lu LY Yu Y. CEO equity incentive duration and expected crash risk. Br Account Rev. (2024) 56:101265. doi: 10.1016/j.bar.2023.101265

[18] Marmot M. The influence of income on health: views of an epidemiologist. Health Aff. (2002) 21:31–46. doi: 10.1377/hlthaff.21.2, 11900185

[19] WHO Commission on Social Determinants of Health. Closing the gap in a generation: health equity through action on the social determinants of health: Commission on Social Determinants of Health final report. World Health Organization (2008).

[20] Kopasker D Montagna C Bender KA. Economic insecurity: a socioeconomic determinant of mental health. SSM Popul Health. (2018) 6:184–94. doi: 10.1016/j.ssmph.2018.09.006, 30417065 PMC6215053

[21] Kárpáti D Renneboog L. Corporate financial frictions and employee mental health. J Financ Quant Anal. (2024) 59:2256–98. doi: 10.1017/S0022109023000595

[22] Kolb RW. Too much is not enough: Incentives in executive compensation. Oxford University Press. (2012). 155–162. doi: 10.1093/acprof:osobl/9780199829583.001.0001

[23] Andreou PC Louca C Petrou AP. CEO age and stock price crash risk. Rev Financ. (2017) 21:1287–325. doi: 10.1093/rof/rfw056

[24] Reichmann D. Tone management and stock price crash risk. Journal of Accounting and Public Policy. (2023) 42:107155–445. doi: 10.1016/j.jaccpubpol.2023.107155, 38826717

[25] Ferris SP Kumar R Sant R Sopariwala PR. An agency analysis of the effect of long-term performance plans on managerial decision making. Q Rev Econ Finance. (1998) 38:73–91. doi: 10.1016/S1062-9769(99)80105-7

[26] Wruck KH Wu Y. The relation between CEO equity incentives and the quality of accounting disclosures: new evidence. J Corp Finan. (2021) 67:101895. doi: 10.1016/j.jcorpfin.2021.101895

[27] He G Bai L Ren HM. Analyst coverage and future stock price crash risk. J Appl Acc Res. (2019) 20:63–77. doi: 10.1108/JAAR-09-2017-0096

[28] Ali MJ Azam MS Baghdadi G Hasan MM Puwanenthiren P. Analyst career concerns and stock price crash risk. Int Rev Financ Anal. (2025) 104785. doi: 10.1016/j.irfa.2025.104785

[29] Fu R Gao F Kim YH Qiu B. Performance volatility, information availability, and disclosure reforms. J Bank Financ. (2017) 75:35–52. doi: 10.1016/j.jbankfin.2016.11.011

[30] Roszkowska-Menkes M Aluchna M Kamiński B. True transparency or mere decoupling? The study of selective disclosure in sustainability reporting. Crit Perspect Account. (2024) 98:102700. doi: 10.1016/j.cpa.2023.102700

[31] Blankespoor E deHaan E Marinovic I. Disclosure processing costs, investors’ information choice, and equity market outcomes: a review. J Account Econ. (2020) 70:101344. doi: 10.1016/j.jacceco.2020.101344

[32] Healy PM Palepu KG. Information asymmetry, corporate disclosure, and the capital markets: a review of the empirical disclosure literature. J Account Econ. (2001) 31:405–40. doi: 10.1016/S0165-4101(01)00018-0

[33] Tang Y. Information disclosure and price discovery. J Financ Mark. (2014) 19:39–61. doi: 10.1016/j.finmar.2014.03.002

[34] Dimson E. Risk measurement when shares are subject to infrequent trading. J Financ Econ. (1979) 7:197–226. doi: 10.1016/0304-405X(79)90013-8

[35] Granados JAT. Recessions and mortality in Spain, 1980–1997. Eur J Population. (2005) 21:393–422. doi: 10.1007/s10680-005-4767-9

[36] Hoepner AG Oikonomou I Sautner Z Starks LT Zhou XY. ESG shareholder engagement and downside risk. Rev Financ. (2024) 28:483–510. doi: 10.1093/rof/rfad034

[37] Lee S Lee S Ryu JY. Do competent managers hoard bad news? Self-regulation theory and Korean evidence. Financ Res Lett. (2021) 41:101836. doi: 10.1016/j.frl.2020.101836

[38] Rösch DM Subrahmanyam A van Dijk MA. Investor short-termism and real investment. J Financ Mark. (2022) 59:100645. doi: 10.1016/j.finmar.2021.100645

[39] Stein JC. Efficient capital markets, inefficient firms: a model of myopic corporate behavior. Q J Econ. (1989) 104:655–69. doi: 10.2307/2937861

[40] Nickell S. Biases in dynamic models with fixed effects. Econometrica. (1981) 49:1417–26. doi: 10.2307/1911408

