# Supplementary material for: Managerial short-termism, firm-level financial instability, and socioeconomic determinants of health: evidence from Chinese listed firms
Source: Front Public Health. 2026 Jul 23;14:1826827. doi: 10.3389/fpubh.2026.1826827 (PMC13441860; doi:10.3389/fpubh.2026.1826827)
Supplement: Supplementary file 1 [file Table_1.docx]

**Appendix Tables**

Table A1. Full baseline regression results

| **Variables** | **Baseline** | **Dynamic** |
| --- | --- | --- |
| REM | 0.184***  (4.21) | 0.137***  (3.46) |
| Lagged stock price crash risk | — | 0.293***  (6.17) |
| Firm size | -0.021**  (-2.34) | -0.018**  (-2.11) |
| Leverage | 0.067***  (3.01) | 0.055**  (2.45) |
| ROA | -0.142***  (-4.07) | -0.119***  (-3.58) |
| Market-to-book (MTB) | 0.012*  (1.78) | 0.009*  (1.65) |
| Volatility | 0.045***  (3.12) | 0.038***  (2.89) |
| Turnover | 0.021**  (2.11) | 0.018*  (1.76) |
| Firm age | -0.004*  (-1.72) | -0.003  (-1.45) |
| Analyst coverage | -0.006*  (-1.89) | -0.005*  (-1.67) |
| Firm FE | Yes | Yes |
| Year FE | Yes | Yes |
| Clustered SE (firm level) | Yes | Yes |
| Adjusted R2 | 0.261 | 0.338 |
| Observations | 27,856 | 27,856 |

Notes: The dependent variable is NCSKEW (t+1). REM is measured in year t. All control variables listed in Section 3.3 are included in the regressions. All coefficients are reported in the table. All regressions include firm and year fixed effects. Standard errors are clustered at the firm level. t-statistics are reported in parentheses. ***, **, and * denote significance at the 1%, 5%, and 10% levels. Variable units follow Table 1: REM is a unit-free standardized composite index; CEO Incentive Duration is in years; Analyst Coverage is in number of analysts.

Table A2. Full moderation regression results

| **Variables** | **Duration model** | **Analyst model** |
| --- | --- | --- |
| REM | 0.203***  (4.56) | 0.176***  (3.98) |
| Duration | -0.041***  (-3.72) |  |
| REM × Duration | -0.028**  (-2.19) |  |
| Analyst coverage |  | -0.014**  (-2.21) |
| REM × Analyst |  | -0.009*  (-1.78) |
| Firm size | -0.019**  (-2.08) | -0.017*  (-1.86) |
| Leverage | 0.061**  (2.48) | 0.058**  (2.33) |
| ROA | -0.131***  (-3.91) | −0.128***  (−3.84) |
| Market-to-book (MTB) | 0.011*  (1.71) | 0.010  (1.69) |
| Volatility | 0.043***  (3.05) | 0.041***  (2.94) |
| Turnover | 0.019**  (1.98) | 0.017*  (1.81) |
| Firm age | −0.004  (−1.69) | −0.003  (−1.41) |
| Firm FE | Yes | Yes |
| Year FE | Yes | Yes |
| Clustered SE (firm level) | Yes | Yes |
| Adjusted R2 | 0.297 | 0.281 |
| Observations | 27,856 | 27,856 |

Notes: The dependent variable is NCSKEW (t+1). REM is measured in year t. Duration captures CEO incentive horizon, and Analyst coverage captures external monitoring. All control variables listed in Section 3.3 are included in the regressions. All coefficients are reported in the table. All regressions include firm and year fixed effects. Standard errors are clustered at the firm level. t-statistics are reported in parentheses. ***, **, and * denote significance at the 1%, 5%, and 10% levels. Variable units follow Table 1: REM is a unit-free standardized composite index; CEO Incentive Duration is in years; Analyst Coverage is in number of analysts.

Table A3. Change-on-change model (NCSKEW)

| **Variables** | **Change-on-change model** |
| --- | --- |
| Change in managerial short-termism (ΔREM) | 0.118***  (3.24) |
| Change in firm size | -0.011*  (-1.89) |
| Change in leverage | 0.034**  (2.21) |
| Change in return on assets | -0.091***  (-3.47) |
| Firm fixed effects | No |
| Year fixed effects | Yes |
| Clustered SE (firm level) | Yes |
| Adjusted R² | 0.112 |
| Observations | 25,800 |

Notes: The dependent variable is the change in stock price crash risk measured by negative conditional skewness (NCSKEW). All independent variables are expressed in first differences. ΔREM denotes the change in managerial short-termism proxied by real earnings management. Year fixed effects are included to absorb common time-varying shocks. Firm fixed effects are not included because first differencing removes time-invariant firm-specific effects. Standard errors are clustered at the firm level. t-statistics are reported in parentheses. ***, **, and * denote significance at the 1%, 5%, and 10% levels, respectively.

Table A4. Change-on-change model (DUVOL)

| **Variables** | **Change-on-change model** |
| --- | --- |
| Change in managerial short-termism (ΔREM) | 0.094***  (2.98) |
| Change in firm size | -0.008*  (-1.71) |
| Change in leverage | 0.029**  (2.02) |
| Change in return on assets | -0.076***  (-3.05) |
| Firm fixed effects | No |
| Year fixed effects | Yes |
| Clustered SE (firm level) | Yes |
| Adjusted R² | 0.096 |
| Observations | 25,800 |

Notes: The dependent variable is the change in stock price crash risk measured by down-to-up volatility (DUVOL). All independent variables are expressed in first differences. ΔREM denotes the change in managerial short-termism proxied by real earnings management. Year fixed effects are included to absorb common time-varying shocks. Firm fixed effects are not included because first differencing removes time-invariant firm-specific effects. Standard errors are clustered at the firm level. t-statistics are reported in parentheses. ***, **, and * denote significance at the 1%, 5%, and 10% levels, respectively.

Table A5. Change-on-change model using alternative crash-risk proxy

| **Variables** | **Change-on-change model** |
| --- | --- |
| Change in managerial short-termism (ΔREM) | 0.103***  (3.12) |
| Change in firm size | -0.016*  (-1.83) |
| Change in leverage | 0.047**  (2.26) |
| Change in return on assets | -0.128***  (-3.64) |
| Firm fixed effects | No |
| Year fixed effects | Yes |
| Clustered SE (firm level) | Yes |
| Adjusted R² | 0.108 |
| Observations | 25,800 |

Notes: This table reports first-difference regressions using an alternative stock price crash risk proxy as the dependent variable. The dependent variable and all independent variables are expressed in first differences. ΔREM denotes the change in managerial short-termism proxied by real earnings management. Year fixed effects are included to absorb common time-varying shocks. Firm fixed effects are not included because first differencing removes time-invariant firm-specific effects. Standard errors are clustered at the firm level. t-statistics are reported in parentheses. ***, **, and * denote significance at the 1%, 5%, and 10% levels, respectively.

Table A6. Supplementary instrumental-variable analysis

| **Variables** | **2SLS Estimation** |
| --- | --- |
| **Panel A: First-stage results (Dependent variable: REM)** | |
| Industry-year average REM (instrument) | 0.483***  (8.14) |
| Firm size | −0.021**  (−2.18) |
| Leverage | 0.044**  (2.09) |
| Return on assets | −0.112***  (−3.31) |
| Firm fixed effects | Yes |
| Year fixed effects | Yes |
| First-stage F-statistic | 66.24 |
| **Panel B: Second-stage results (Dependent variable: NCSKEW t+1)** | |
| Instrumented REM | 0.136***  (3.08) |
| Firm size | −0.018*  (−1.94) |
| Leverage | 0.051**  (2.17) |
| Return on assets | −0.139***  (−3.52) |
| Firm fixed effects | Yes |
| Year fixed effects | Yes |
| Adjusted R² | 0.223 |
| Observations | 27,856 |

Notes: This table reports two-stage least squares (2SLS) estimates. Panel A presents the first-stage results, where firm-level REM is regressed on the industry-year average REM (excluding the focal firm) used as the instrument. The first-stage F-statistic of 66.24 exceeds the conventional threshold of 10, indicating strong instrument relevance (Staiger & Stock, 1997). Panel B presents the second-stage results, where the fitted REM is used to explain subsequent stock price crash risk (NCSKEW t+1). All models include firm and year fixed effects. Firm-level clustered t-statistics are reported in parentheses. ***, **, and * denote significance at the 1%, 5%, and 10% levels, respectively. Variable units follow Table 1: REM is a unit-free standardized composite index; CEO Incentive Duration is in years; Analyst Coverage is in number of analysts. The first-stage F-statistic indicates instrument relevance only and should not be interpreted as evidence of instrument exogeneity. Because industry-year shocks may directly affect crash risk, the IV results are interpreted as supportive robustness evidence rather than definitive causal evidence.

Table A7. Heterogeneity Test: Corporate Governance Quality

| **Variables** | **Heterogeneity model** |
| --- | --- |
| Managerial short-termism (REM) | 0.143***  (3.41) |
| High corporate governance | -0.029**  (-2.07) |
| REM×High corporate governance | -0.021*  (-1.86) |
| Firm fixed effects | Yes |
| Year fixed effects | Yes |
| Adjusted R² | 0.254 |
| Observations | 27,856 |

Notes: This table examines heterogeneity based on corporate governance quality. High corporate governance is defined as firms with governance measures above the sample median. The interaction term tests whether stronger governance mitigates the effect of managerial short-termism on stock price crash risk. Firm and year fixed effects are included. t-statistics are reported in parentheses. ***, **, and * denote significance at the 1%, 5%, and 10% levels, respectively.

Table A8. Heterogeneity Test: Essential-Service Industries

| **Variables** | **Heterogeneity model** |
| --- | --- |
| Managerial short-termism (REM) | 0.168***  (3.87) |
| Essential-service industry | 0.024*  (1.73) |
| REM×Essential-service industry | 0.036**  (2.14) |
| Firm fixed effects | Yes |
| Year fixed effects | Yes |
| Adjusted R² | 0.238 |
| Observations | 27,856 |

Notes: This table examines heterogeneity across essential-service industries. Essential-service industries include healthcare services, pharmaceuticals, food supply, and transportation. These sectors are more closely related to employment stability, supply continuity, and access to essential goods and services. The interaction term tests whether the association between managerial short-termism and stock price crash risk is more pronounced in industries with broader socioeconomic relevance. Firm and year fixed effects are included. t-statistics are reported in parentheses. ***, **, and * denote significance at the 1%, 5%, and 10% levels, respectively.

Table A9. Classification of essential-service industries

| **Category** | **Included sectors** |
| --- | --- |
| Healthcare services | Hospitals, medical services, healthcare providers |
| Pharmaceuticals | Drug manufacturing, biotechnology firms |
| Food supply | Food production, processing, and distribution |
| Transportation | Logistics, freight, and public transport systems |

Notes: Industries are classified as essential-service industries if they are closely related to employment stability, supply continuity, access to essential goods and services, or the functioning of health-related and socioeconomic infrastructure.

Notes: This figure plots the marginal effect of REM on NCSKEW across the observed range of CEO incentive duration, based on the coefficients reported in Table 4.

Figure A1. Marginal effect of REM on future stock price crash risk under different levels of CEO incentive duration.

Notes: This figure plots the marginal effect of REM on NCSKEW across the observed range of analyst coverage, based on the coefficients reported in Table 5.

Figure A2. Marginal effect of REM on future stock price crash risk under different levels of analyst coverage.
